# Limiting the gamble: Risk and predictability for renal replacement therapy in patients receiving mechanical circulatory support★

**DOI:** 10.1051/ject/2024041

**Published:** 2025-03-07

**Authors:** Kelsey Gore, Dean Linder, Juan José Martinez Duque, Junxi Wang, Brett Wester, Tiffany Otero, Shaun Yockelson, Adrian Alexis Ruiz, Bobby D. Nossaman

**Affiliations:** 1 Department of Cardiovascular Perfusion and Extracorporeal Technology, Ochsner Health 1514 Jefferson Highway New Orleans Louisiana 70121 USA; 2 CES University Cl 10A #22-04, El Poblado Medellin Antioquia Columbia; 3 The University of Queensland Medical School 288 Herston Road Herston QLD 4006 Australia; 4 Critical Care Section, Anesthesiology & Perioperative Medicine, Ochsner Health 1514 Jefferson Highway New Orleans Louisiana 70121 USA

**Keywords:** Extracorporeal membrane oxygenation (ECMO), Renal replacement therapy (RRT), Mechanical circulatory support (MCS), Anticoagulation, Inflammation

## Abstract

*Background*: Patients receiving mechanical circulatory support (MCS) frequently require renal replacement therapy (RRT). Examining risk factors for requiring RRT in patients receiving MCS may allow improved understanding of these comorbidities and enhance patient outcomes. *Methods*: Following IRB approval, patient characteristics, comorbidities, and the need for RRT were studied in 129 patients who received MCS from January 2017 to October 2023. The clinical variables underwent machine learning to examine their relationships to the outcome of interest, the need for RRT. *Results*: In this study, the incidence of RRT was 36% with a 95% confidence interval ranging from 29% to 44%. Following machine learning, patients with a history of immunologic therapy or having a pacemaker or internal cardiac defibrillator (ICD) were associated with the need for RRT (*χ*^2^ = 44, *P* = 0.0003). The c-index statistic for this model was 0.81. The anticoagulation therapy administered in these two groups was also analyzed. Patients in these two groups receiving unfractionated heparin were observed to have a higher incidence (44%) in the need for RRT. *Conclusion*: The incidence of RRT was high in this patient population. The novel associations in patients requiring MCS who have received prior immunologic therapy or have pre-existing pacemaker/ICDs suggest that an increased systemic inflammatory state exists that escalates the need for RRT. Unfractionated heparin appears to provide minimal protection from the need for RRT in patients requiring MCS. These findings suggest that other options for systemic anticoagulation in patients requiring MCS should be considered. Further investigation into how these background inflammatory conditions contribute to the need for RRT in patients requiring MCS is warranted.

## Introduction

Cardiopulmonary shock contributes to the development of end-organ hypoperfusion [1]. Although the therapy for this type of shock includes the administration of intravenous vasoactive medications, percutaneous coronary artery interventions, and/or mechanical ventilation, patients who continue to deteriorate may require mechanical circulatory support (MCS) [2]. MCS swiftly augments tissue perfusion and assists to normalize the pathophysiology observed in these conditions [2].

Although the reperfusion of tissues with MCS is beneficial, this therapy risks development of reperfusion injury due to the prior hypoperfusion period as well as due to further development of reactive oxygen/nitrogen species, cytokines release, and hyperinflammatory responses exacerbating tissue injury [2, 3]. Acute kidney injury frequently develops in patients requiring MCS [3–5]. However, the etiologies for the need of RRT during MCS are unclear [6, 7]. The purpose of this investigation was to examine the association of patient comorbidities receiving MCS with the need for RRT.

## Materials and methods

Following IRB approval, a retrospective analysis of patient characteristics, comorbidities, and the incidence of RRT were studied from January 2017 to October 2023 in 129 patients receiving 159 MCS devices (Table 1) at Ochsner Health-Jefferson Highway Campus in New Orleans, Louisiana. There were no patient exclusion criteria. Patient characteristics and recorded comorbidities (Table 2) underwent machine learning to determine associations in the need for RRT [8].

**Table 1:** List of mechanical circulatory support devices.

Device	Count	%
ECMO	96	60.4
IABP	29	18.2
Impella	11	6.9
VAD	23	14.5
Total	159	100.0

**Table 2:** Baseline characteristics and reported comorbidities for renal replacement therapy in patients requiring mechanical circulatory support.

Terms	Estimates	Std Error	*χ*^2^	*P* values
Intercept	3.7	1.6	5.7	0.0171
Age	−0.07	0.02	12.5	0.0004*
Sex, female	−0.04	0.26	2.5	0.1159
BMI	−0.01	0.04	0.2	0.6909
Insulin-dependent diabetes	1.4	1.4	1.0	0.3194
Chronic renal failure	0.3	0.3	0.5	0.4608
Chronic cardiovascular disease	0.4	0.3	1.5	0.2261
Immunomodulation	0.8	0.3	7.3	0.0067*
Structural lung disease	−0.3	0.3	0.9	0.3343
Pacemaker/Internal cardiac defibrillator	0.6	0.3	4.2	0.0411*
Atrial fibrillation	0.3	0.3	1.4	0.2430
Endocarditis	0.4	0.7	0.3	0.5936
Previous cardiac surgery	0.3	0.2	0.9	0.3357
Congestive heart failure	−0.6	0.3	3.0	0.0845
Peripheral vascular disease	−0.02	0.5	0	0.9611

(*) Denotes the baseline characteristics and comorbidities associated with the outcome of interest, the need for renal replacement therapy, are statistically significant.

### Statistics

Baseline characteristics and comorbidities (Table 2) underwent machine learning to explore these relationships for the need of RRT. The machine learning used in this prediction study included Decision-Tree (Recursive Partitioning), Bootstrap Forest, Boosted Tree, K Nearest Neighbors, Neural Support Vectors Machines, Discriminant, Fit Least Squares, Fit Stepwise, Logistic Regressions, Generalized Regression, Native Bayes, and Partial Least Squares [8]. *P* values for frequentist tests were set for statistical significance at <0.05. The statistical program, JMP^®^ Pro 17.2 (SAS Institute, Cary, NC) was utilized for this study [8].

## Results

In this study of patients requiring MCS, the incidence of RRT was 36% with a 95% confidence interval (CI) 29–44%. The incidence of hospital mortality in patients requiring RRT was 79% CI 66.7–87.5% (*χ*^2^ = 29, *P* < .0001) but was 35.3% CI 26.7–44.9% in MCS patients not requiring RRT. The types of MCS devices used in this study are shown in Table 1. Baseline patient characteristics and recorded comorbidities in patients requiring MCS are shown in Table 2. The baseline characteristics and comorbidities underwent machine learning associated with the outcome of interest, the need for RRT. Age, and two novel comorbidities, patients with a history immunomodulation, and patients with pacemaker/internal cardiac defibrillator (ICD) were statistically associated with the need for RRT (*χ*^2^ = 44, *P* = 0.0003; Table 2). The c-index statistic for this model was 0.81. Based on the results of this model, two contingency tables were constructed to further explore the two novel comorbidities with the need for RRT (Tables 3 and 4). Patients who had a history of immunomodulation were noted to have an incidence of 48% in the need for RRT during MCS (Table 3). Patients with a history of pacemaker/ICDs also had a high incidence (47%) in requiring RRT during MCS (Table 4). Patients with both comorbidities had a 66% incidence in the need for RRT.

**Table 3:** Contingency table of the association of renal replacement therapy in patients with a history of immunomodulation during mechanical circulatory support.

		Renal replacement therapy		
	Counts (%)	Yes	No	Total
Immunomodulation	Yes	12 (48)	13 (52)	25
	No	45 (34)	89 (66)	134
	Total	57	102	159

**Table 4:** Contingency table of the association of renal replacement therapy in patients with pre-existing pacemaker or internal cardiac defibrillator (ICD) during mechanical circulatory support.

		Renal replacement therapy		
	Counts (%)	Yes	No	Total
Pacemaker/ICD	Yes	25 (47)	28 (53)	53
	No	32 (30)	74 (70)	106
	Total	57	102	159

We further explored the role of anticoagulation used in the two novel groups when combined (Interest groups) and the results of that analysis are shown in Table 5. In MCS patients receiving unfractionated heparin (UFH), a 43% incidence in the need for RRT was observed in this cohort of patients. Four patients who did not receive anticoagulation therapy all required RRT, in contrast to three patients not requiring RRT when low molecular weight heparin (LMWH) was used (*χ*^2^ = 10.1, *P* = 0.0064). While these findings were observed in a small subset of patients, the results warrant further investigation into anticoagulation practices used in this patient population.

**Table 5:** Contingency table of the association of renal replacement therapy by anticoagulant therapy during mechanical circulatory support.

		Renal replacement therapy		
	Counts (%)	Yes	No	Total
Interest groups	UFH	26 (43)	35 (57)	61
	LMWH	0 (0)	3 (100)	3
	None	4 (100)	0 (0)	4
	Total	30	38	68

## Discussion

The use of MCS therapies is becoming an important component of supportive care in intensive care units [5]. Although initial support for patients frequently includes vasoactive support medications and/or mechanical ventilation, patients that continue to deteriorate or become refractory to medical therapy may require MCS [1, 2].

Although the causes of cardiogenic shock are numerous, a low cardiac output state exists, that when unsuccessfully treated, results in end organ hypoperfusion [5]. In our study, we observed two novel preexisting risk factors for the need of RRT during MCS; patients with preexisting inflammatory disorders requiring therapy, and patients with pre-existing pacemaker/ICDs. As we observed a higher incidence in the need for RRT in patients with these two disorders, this association suggests that an increased systemic inflammatory state exists that escalated the need for RRT [7], as in these two groups, the administration of UFH was not protective in reducing the need for RRT.

UFH is frequently used for anticoagulation during MCS which was based upon prior experience in procedures requiring cardiopulmonary bypass [9]. However with long-term UFH administration, heparin resistance and immune-mediated platelet activation leading to heparin-induced thrombocytopenia can develop [10]. In hypercoagulable states, such as observed in patients with renal failure, following major surgery, or histories of congestive heart failure, or autoimmune diseases, Kaur, Arsene, and colleagues recommend UFH should be used with caution [11]. Implantable cardiac devices have also been shown to generate an inflammatory response [12]. Taken together, the inflammatory components in UFH may contribute to the inflammatory state and increase the need for RRT. Studies with newer generation anticoagulants need to be conducted following development of bedside monitoring techniques to allow timely adjustment of anticoagulant therapy based upon real-time coagulation parameters [9, 10, 13].

## Limitations

Limitations of retrospective studies suffer from completeness of medical record data. However, the strength of this study was the near 100% data collection due to the recent development of electronic medical records. Another limitation of this study is potential bias due to confounding. However, the strength of this study was the statistical method used to adjust for all confounders through the application of machine learning against the outcome of interest, need for RRT. Machine learning performs better than traditional statistical analyses, especially when analyzing multifaceted data sets. The ability to utilize machine modeling provides a powerful tool to express information [14].

## Conclusions

The incidence of RRT was high in this patient population. The mortality rate was high in patients requiring RRT. Moreover, these findings also suggest that other options for systemic anticoagulation during MCS should be considered. The novel associations of patients who have received prior immunotherapy or with pre-existing pacemaker/ICDs requiring MCS suggest an increased systemic inflammatory state exists that escalates the need for RRT. Further investigation into how these background inflammatory conditions contribute to the need for RRT during MCS is warranted.

## Data Availability

All available data are incorporated into the article.
